# Exploring the occupational protection potential of an intravenous compounding robot across the cytotoxic drug workflow

**DOI:** 10.3389/fpubh.2026.1901687

**Published:** 2026-08-18

**Authors:** Jing Sun, Bing Liu, Zhiqing Li, Ruoyu Hu, Shujuan Bai

**Affiliations:** 1 Department of Pharmacy Intravenous Admixture Services, Zibo First Hospital, Zibo, Shandong, China; 2 Department of Clinical Pharmacy, Zibo First Hospital, Zibo, Shandong, China

**Keywords:** cytotoxic drugs, entire workflow, intravenous compounding robot, occupational protection, Pharmacy intravenous admixture services, PIVAS

## Abstract

**Objective:**

To evaluate an intravenous compounding robot's occupational protection across the full workflow (compounding, packaging, administration) by measuring cytotoxic drug residues, and to assess its cleaning protocol and cross-contamination risk for subsequent non-cytotoxic drug compounding.

**Methods:**

(1)Questionnaire survey: Cytotoxic drug handlers at various workflow stages were surveyed for occupational protection and awareness. (2) Experimental study: Cyclophosphamide and fluorouracil were selected for a 3-day robotic vs. manual compounding comparison. Surface wipes samples from 168 sites were quantified by HPLC-MS/MS. (3) Recommendations were derived by integrating the questionnaire and experimental results.

**Results:**

The survey revealed that all respondents (100%) acknowledged that their work stage involved an occupational exposure risk and that protective measures were necessary, but only compounding staff strictly adhered to protection, 18.2% of packaging staff, 14.3% of transport workers lacked gloves, while only 45.5% of nurses always wear gloves each time. Experimental data showed that robotic compounding substantially reduced surface residues at compounding sites (gloves and gown sleeves) compared with manual compounding, with manual groups showing sporadic extreme values (e.g., fluorouracil on gloves: 834.340 ng/cm^2^). Residues were also detected on packaging and administration—particularly in the manual group (fluorouracil on bags: up to 734.704 ng/cm^2^)—indicating that exposure risks extend beyond the compounding stage. Internal robotic residues were low, though a single post-cleaning detection of 0.018 ng/cm^2^ suggests minor cleaning blind spots. No detectable cross-contamination was observed when non-cytotoxic drugs were subsequently compounded.

**Conclusions:**

This preliminary study suggests that the intravenous compounding robot shows potential for reducing occupational exposure across compounding, packaging, and administration, with the most pronounced effect observed during compounding. However, exposure risks persist in packaging and administration, particularly in manual operations, and inadequate protective awareness among staff calls for improved supervision and training. Additionally, initial findings indicate that internal contamination after cytotoxic compounding is low and can be further reduced by cleaning, supporting the tentative feasibility of using the same robot for subsequent non-cytotoxic compounding. Cross-contamination risk appears low and controllable, as yet, it has not been possible to translate residue value directly into actual cancer risks for staff, further validation is needed.

## 1 Introduction

Cytotoxic drugs (CDs) inhibit tumor cell proliferation by interfering with cellular nucleic acid metabolism and blocking protein synthesis, serving as the cornerstone of modern tumor chemotherapy regimens (1, 2). However, numerous studies have demonstrated that long-term, low-dose occupational exposure to CDs among healthcare workers can lead to risks such as alopecia, skin irritation, reproductive toxicity, and even carcinogenicity (3–7). Minimizing occupational exposure during the use of CDs is a globally recognized occupational safety concern.

In 2010, China established the Quality Management Standards for Centralized Intravenous Admixture Services, encouraging the adoption of the Pharmacy Intravenous Admixture Services (PIVAS) model and mandating the centralized preparation of hazardous medications. While protection has improved through centralized systems like biological safety cabinets (BSCs), operators still need to manually dissolve drugs, transfer, and aspirate, which makes it challenging to fully prevent aerosol spread, contamination of infusion bags, and needle leaks. Cytotoxic drugs can contaminate the masks and gloves of pharmacy technicians, and this contamination is significantly correlated with oxidative DNA damage in their bodies (8). Furthermore, risks of occupational exposure continue during later stages such as packaging, transportation, and administration of the final product.

In recent years, various advanced compounding technologies have been explored in China and internationally. Studies on intravenous compounding robots have primarily focused on compounding quality, accuracy, user experience, safety and social benefits, with occupational exposure evaluations limited to the compounding room interior (9–17). There remains a critical need to address the assessment of occupational exposure during subsequent packaging and administration processes, as well as to supplement the existing data on cross-contamination risks associated with robotic compounding.

The present study aimed to measure drug residues at operational points throughout the entire workflow, comparing the robot with manual compounding to evaluate occupational exposure at each stage. The specific objectives were as follows: (1) to quantify the direct protective advantage of the robot for compounding personnel; (2) to evaluate the indirect protective effect of the robot on packaging and drug administration personnel; (3) to verify the effectiveness of the robot's cleaning protocol; (4) to evaluate the cross-contamination risk when non-cytotoxic drugs are compounded after CDs, providing data to support safe device operation; and (5) to comprehend the current status of healthcare workers' occupational protection awareness and behavior through a questionnaire survey, revealing potential risks amid the adoption of new technology.

## 2 Materials and methods

### 2.1 Study design

This study employed a mixed-methods design combining a questionnaire survey and an experimental investigation, conducted in the PIVAS of our hospital. The experimental group used an intravenous compounding robot (model: WEIANS Onco PD-160C, manufacturer: Shenzhen Weibond Technology Co., Ltd.) for cytotoxic drug compounding, while the control group used a Class II Type B2 BSC for manual compounding according to standard operating procedures (SOPs). The oprating environment with the requirements of the Guidelines for the Construction and Management of Pharmacy Intravenous Admixture Services issued by the National Heath Commission of China.

To ensure comparability, both groups were operated by the same cohort of pharmacy staff, all of whom had completed standardized training and passed competency-based skills assessments. The compounding procedures were conducted in an identical working environment, on different dates but within equivalent time windows, and each time, only the specific prescription is prepared. All operations were carried out before using the robots and biosafety cabinets for other tasks, to ensure that neither the operators nor the environment would be disturbed. All preparations followed the most commonly prescribed regimens at our institution: cyclophosphamide 0.8 g in 0.9% sodium chloride injection 100 ml, and fluorouracil 0.5 g in 5% glucose injection 250 ml. These doses represent the single-prescription test amounts used consistently throughout the three-day comparative experiment. In the manual compounding group, the preparation strictly followed the standard operating procedures for cytotoxic drug handling, which included:(1)Surface disinfection of the workbench and materials; (2) For cyclophosphamide vials: drawing up diluent with a syringe, puncturing the vial stopper to inject the diluent, agitating to dissolve the powder completely, and withdrawing the drug solution for injection into the infusion bag. For fluorouracil ampoules: puncturing and withdrawing the drug solution with a syringe, and injecting it into the infusion bag; (3) Check the quality of the finished infusion and place it in the transfer window. In the robotic compounding group, The only manual operations required were: (1) surface disinfection of the materials; (2) placing the drug vials/ampoules and empty infusion bags into the designated positions on the robotic platform; and (3) removing the prepared infusion bags after the compounding cycle was completed. Check the quality of the finished infusion and place it in the transfer window.

The questionnaire was newly designed based on an authoritative Chinese guidelines for occupational protection (18) and the local workflow of cytotoxic drug handlers. Content validity was assured through review by a nine-member expert panel. Anonymous administration and explicit instruction that “no standard answers exist” were applied to reduce response bias. All staff participating in the questionnaire survey had received standardized training. The study was divided into three phases: (1) questionnaire survey—to assess occupational exposure awareness and protection status; (2) experimental study—sample collection and analysis; and (3) data analysis and conclusion formulation.

### 2.2 Questionnaire survey

#### 2.2.1 Participants

Staff from clinical departments, PIVAS, pharmaceutical supply, and nursing departments who had contact with CDs, covering the entire workflow. Prior to the initiation of this study, ethical approval was obtained. The questionnaire was conducted after submitting a written application and receiving approval.

#### 2.2.2 Survey contents

An anonymous, self-designed questionnaire was used, covering the following aspects: (1) basic information: age, gender, professional title, position, years of CD exposure, etc.; (2) knowledge about CDs: hazards, exposure routes, protective measures, emergency procedures, etc.; (3) awareness of occupational protection: perceived importance, willingness to implement protective measures; and (4) current status of occupational protection: standardized implementation of protective measures at each stage.

### 2.3 Experimental study

#### 2.3.1 Materials

List of instruments, reagents, and drugs used in this study is shown in Table 1.

**Table 1:** List of instruments, reagents, and drugs used in this study.

Type	Product	Model	Manufacturer/brand
Equipment	Intravenous compounding robot	WEINAS Onco PD-160M (C)	Shenzhen Weibond Technology Co., Ltd.
	Biological safety cabinet	BSC-1300IIA2	Shanghai Shangjing Purification Equipment Co., Ltd.
	HPLC-MS/MS	TSQ Quantiva	Thermo Fisher Scientific Inc
	Ultrasonic cleaner	KQ-800DE	Kunshan Ultrasonic Instrument Co., Ltd.
	Vortex mixer	XW-80A	Haimen KYlin-Bell Lab Instruments Co., Ltd
	Centrifuge	TGL-16M	Hunan Xiangyi Laboratory Instrument Development Co., Ltd
**–**	**Product**	**Grade**	**Manufacturer/brand**
Reagent	Acetonitrile	HPLC grade	Chengdu Kelong Chemical Co., Ltd
	Methanol	HPLC grade	Merck & Co., Inc
	Formic acid	HPLC grade	Shanghai Macklin Biochemical Co., Ltd
	Isopropanol	HPLC grade	ANPEL Laboratory Technologies Inc
	Ultrapure water	UP	/
	**Product**	**Strength**	**Manufacturer/brand**
Drug	Fluorouracil injection	0.5 g:10 mL	Qilu Pharmaceutical Co., Ltd.
	Cyclophosphamide for injection (Endoxan)	0.2 g	Baxter oncology GmbH

#### 2.3.2 Sample collection

**Figure 1:**
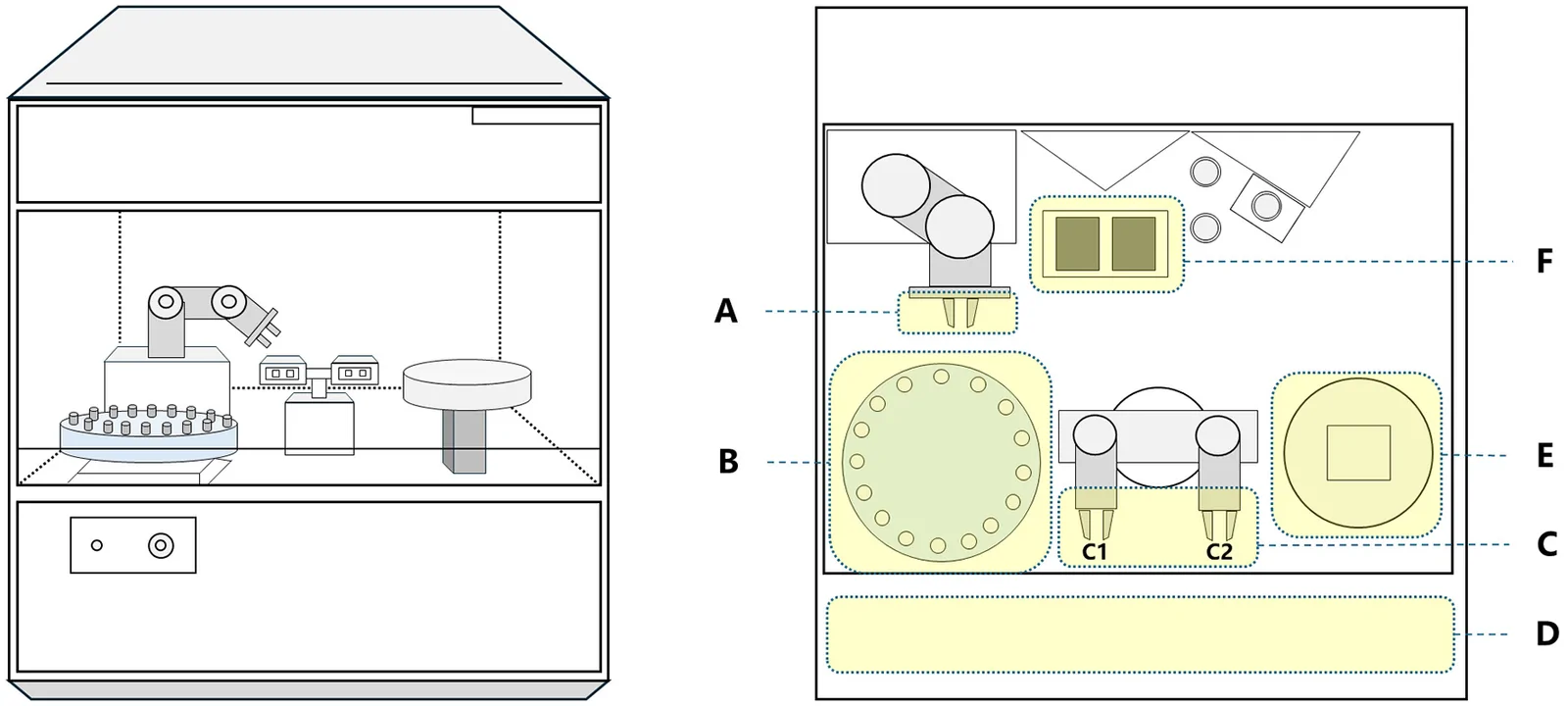
Schematic diagram of internal sampling sites within the intravenous compounding robot.

Sampling sites included personnel contact points during the compounding, packaging, and administration stages, as well as various sites inside the robot, a total of 56 fixed sampling sites (28 for the robotic group and 28 for the manual group), were sampled once daily for three consecutive days (168 samples in total). All surface samples were collected using the wipe method. Cotton swabs moistened with methanol were used to wipe a defined area (10 cm × 10 cm) in an S-shaped pattern, with overlapping strokes and consistent pressure applied throughout the process. Detailed sampling site design is shown in Tables 2, 3, and Figure 1.

**Table 2:** Sampling sites for the compounding, packaging, and administration stages.

Stage	Sampling area	Robotic group (WEINAS)	Manual group (BSC)
Compounding	Gloves (palm)	A1	A2
	Gown (sleeve surface)	B1	B2
	Gown (chest surface)	Gown B1-1	Gown B2-1
	Mask (outer surface)	D1	D2
Packaging and Administration	Infusion bag surface at immediate post-compounding packaging	A1	A2
	Workbench surface where bags were placed after unpacking (60 min later)	B1	B2

**Table 3:** Sampling sites inside the robot.

Sampling site	Sampling area	Before cleaning	After cleaning
Inside robot	Drug gripper finger (site A)	A1	A2
	Drug loading station (site B)	B1	B2
	Consumable loading gripper (site C)—drug side	C1	C2
	Consumable loading gripper (site C)—diluent side	C2–1	C2–2
	Drug preparation table (site D)	D1	D2
	Diluent loading position (site E)	E1	E2
	Waste bin outlet (site F)	F1	F2
Non-cytotoxic infusion after cytotoxic drug compounding	Sampling area	First bag	Last bag
	Finished infusion bag surface (site G)	G1	G2

### 2.4 Sample analysis

#### 2.4.1 Sample pretreatment

The sampling swab was placed into 3 mL of methanol, vortex for 10 min, let the sample stand overnight at 4 °C, and centrifuged at 12,000 r/min for 10 min, collected the supernatant, blown dry with nitrogen gas, reconstituted with 0.2ml of methanol, filtered through a 0.22 μm organic membrane and then analyzed by LC-MS/MS.

#### 2.4.2 Chromatographic and mass spectrometric conditions

Chromatographic separation was performed on an Agilent C18 column (2.1 × 100 mm, 1.7 μm). Mobile phase A was 0.1% formic acid in water, and mobile phase B was acetonitrile. The column temperature was maintained at 30 °C, the injection volume was 2 μl, and the flow rate was 0.3 ml/min. The gradient elution program was as follows: 0–1 min, 95% A; 1–1.5 min, linear decrease to 30% A; 1.5–3.0 min, further decrease to 10% A and held until 4.0 min; 4.0–4.1 min, rapid return to 95% A and equilibration until 5.0 min. Mass spectrometric detection employed an electrospray ionization (ESI) source in multiple reaction monitoring (MRM) mode. Fluorouracil was detected in negative ion mode, monitoring the transition m/z 129 → 59, with a capillary voltage of 3.0 kV, fragmentor voltage of 49 V, and collision energy of 28 eV. Cyclophosphamide was detected in positive ion mode, monitoring the transition m/z 261.1 → 106, with a capillary voltage of 4.0 kV, fragmentor voltage of 61 V, and collision energy of 22 eV. The ion transfer tube temperature was set to 325 °C, the vaporizer temperature to 350 °C, and the sheath gas and auxiliary gas flow rates were 40 Arb and 10 Arb, respectively.

#### 2.4.3 Method validation

The analytical method used in this study was fully validated, and the results are shown in Table 4. These results indicate that the method has good system suitability, specificity, accuracy, and precision, and is suitable for the determination of fluorouracil and cyclophosphamide.

**Table 4:** Method validation results.

Validation parameter	Fluorouracil	Cyclophosphamide	Acceptance criteria
Limit of quantitation (LOQ)	10.00 μg/L	1.00 μg/L	S/N ≥ 10
Limit of detection (LOD)	3.00ug/L	0.50ug/L	S/N ≥ 3
Linear range	10–1,000 μg/L	1–20 μg/L	R^2^ ≥ 0.99
Calibration equation	*y* = 7.752x + 0.000	*y* = 1657.848x + 0.000	/
Correlation coefficient (R^2^)	0.9993	0.9966	≥ 0.99
Recovery	93.07%−101.05%	102.27%−105.36%	80%−120%
Precision (RSD)	0.41%	0.94%	≤ 10%
System suitability (RSD)	0.83%	6.92%	≤ 10%

#### 2.4.4 Data processing and statistical analysis

##### 2.4.4.1 Data processing

The limit of detection (LOD) was 0.006ng/cm^2^ (3.00ug/L) for fluorouracil and 0.001ng/cm^2^ (0.50ug/L) for cyclophosphamide. For samples with concentrations below the LOD, a conservative substitution strategy was adopted, assigning them a value of LOD/2 (i.e., 0.003 ng/cm^2^ for fluorouracil and 0.0005 ng/cm^2^ for cyclophosphamide)—a strategy intended to minimize bias due to data censoring while aligning with standard practices in environmental and occupational exposure monitoring.

Given that the majority of sample measurements fell below these detection limits, and that the sample size per group was limited (*n* = 3 per group), the findings are predominantly reported using descriptive statistical measures, including median, range, and maximum values.

## 3 Results

### 3.1 Questionnaire survey results

#### 3.1.1 Basic information

A total of 36 valid questionnaires were collected, covering multiple positions including PIVAS pharmacists, clinical healthcare workers, and transport personnel. Among the respondents, 11 were nurses (30.6%), 11 were pharmacists (30.6%), 5 were physicians (13.9%), 7 were transport workers (19.4%), and 2 were from other positions (5.6%). Additionally, 86.1% of the respondents had more than 5 years of work experience, and 77.8% were in the 31–50 age range.

#### 3.1.2 Protection awareness

Among the respondents, 88.9% reported having received regular training on cytotoxic drug spill management, 83.3% underwent regular occupational health examinations, and 42.9% had experienced a drug spill or breakage incident. All respondents (100%) believed that their own work stage posed an occupational exposure risk and that protective measures should be taken.

Among PIVAS compounding staff, 27.3% worried that centralized compounding increased their personal occupational exposure. However, 97.2% of respondents believed that the intravenous compounding robot reduced hazards to compounding personnel compared with manual compounding, and 88.9% thought that using the robot was beneficial for occupational protection at their own stage (including compounding, packaging, transport, and administration).

#### 3.1.3 Current protection status

Although 77.8% of the respondents were exposed to CDs weekly or even daily, only the compounding staff (100%) adopted complete protective measures. During finished infusion packaging, 18.2% of the workers did not wear gloves and 36.4% did not wear masks. During ward drug administration, nurses routinely wore masks, but only 45.5% always wear gloves each time. During transport, 14.3% of transport workers did not wear masks or gloves.

### 3.2 Experimental study results

#### 3.2.1 Protective advantage of the robot compared with manual compounding

The occupational exposure levels of the intravenous compounding robot vs. manual compounding were compared, and the detailed results are shown in Tables 5, 6.

**Table 5:** Comparison of operator exposure levels (cyclophosphamide).

Sampling site	Group	Day 1 (ng/cm^2^)	Day 2 (ng/cm^2^)	Day 3 (ng/cm^2^)	Median (ng/cm^2^)	Range (ng/cm^2^)	Maximum (ng/cm^2^)
Gloves (palm)	Robotic	0.0005	0.0005	0.0005	0.0005	0.0005 ~ 0.0005	0.0005
	Manual	0.20190	0.0005	0.0005	0.0005	0.0005 ~ 0.20190	0.20190
Gown (sleeve)	Robotic	0.0005	0.0005	0.0005	0.0005	0.0005 ~ 0.0005	0.0005
	Manual	0.00559	8.44376	0.0005	0.00559	0.0005 ~ 8.44376	8.44376
Gown (chest)	Robotic	0.0005	0.0005	0.0005	0.0005	0.0005 ~ 0.0005	0.0005
	Manual	0.00116	0.0005	0.0005	0.0005	0.0005 ~ 0.00116	0.00116
Mask (outer surface)	Robotic	0.0005	0.0005	0.0005	0.0005	0.0005 ~ 0.0005	0.0005
	Manual	0.0005	0.0005	0.0005	0.0005	0.0005 ~ 0.0005	0.0005

LOD = 0.001 ng/cm^2^. Values below the detection limit are all assigned as LOD/2 = 0.0005 ng/cm^2^.

**Table 6:** Comparison of operator exposure levels (fluorouracil).

Sampling site	Group	Day 1 (ng/cm^2^)	Day 2 (ng/cm^2^)	Day 3 (ng/cm^2^)	Median (ng/cm^2^)	Range (ng/cm^2^)	Maximum (ng/cm^2^)
Gloves (palm)	Robotic	0.003	0.003	0.05554	0.003	0.003 ~ 0.05554	0.05554
	Manual	0.003	10.82062	834.34000	10.82062	0.003 ~ 834.34000	834.34000
Gown (sleeve)	Robotic	0.003	0.003	0.003	0.003	0.003 ~ 0.003	0.003
	Manual	0.003	0.003	0.003	0.003	0.003 ~ 0.003	0.003
Gown (chest)	Robotic	0.003	0.003	0.003	0.003	0.003 ~ 0.003	0.003
	Manual	0.003	0.003	0.003	0.003	0.003 ~ 0.003	0.003
Mask (outer surface)	Robotic	0.003	0.003	0.003	0.003	0.003 ~ 0.003	0.003
	Manual	0.003	0.003	0.003	0.003	0.003 ~ 0.003	0.003

LOD = 0.006 ng/cm^2^. Values below the detection limit are all assigned as LOD/2 = 0.003 ng/cm^2^.

For cyclophosphamide, at the gloves (palm) site, all three measurements in the robotic group were below the detection limit (assigned as 0.0005 ng/cm^2^), with a median of 0.0005 ng/cm^2^ and a range of 0.0005–0.0005 ng/cm^2^. In the manual group, the median was also 0.0005 ng/cm^2^; however, a higher value (0.20190 ng/cm^2^) was observed on Day 1, with a maximum detected value of 0.20190 ng/cm^2^. At the gown (sleeve) site, all samples in the robotic group were below the detection limit. In contrast, the manual group showed a median of 0.00559 ng/cm^2^ and a range of 0.0005–8.44376 ng/cm^2^, with an extreme outlier of 8.44376 ng/cm^2^ on Day 2, suggesting a risk of occasional severe contamination during manual operations at this site. At the gown (chest) site, the robotic group again remained below the detection limit. The manual group exhibited a median of 0.0005 ng/cm^2^ with a range of 0.0005–0.00116 ng/cm^2^, with only Day 1 showing a value slightly above the detection limit (0.00116 ng/cm^2^), indicating generally negligible residual levels. At the mask (outer surface) site, all measurements in both the robotic and manual groups were below the detection limit, with no detectable residues observed in either group.

For fluorouracil, at the gloves (palm) site, the robotic group showed values below the detection limit (0.003 ng/cm^2^) on Days 1 and 2, with a slightly elevated value (0.05554 ng/cm^2^) on Day 3, yielding a median of 0.003 ng/cm^2^ and a range of 0.003–0.05554 ng/cm^2^. In the manual group, Day 1 remained below the detection limit, while Days 2 and 3 exhibited extremely high values of 10.82062 ng/cm^2^ and 834.34000 ng/cm^2^, respectively, with a median of 10.82062 ng/cm^2^ and a range of 0.003–834.34000 ng/cm^2^, indicating a substantial risk of occasional severe contamination during manual operations at this site. At the gown (sleeve), gown (chest), and mask (outer surface) sites, all measurements in both the robotic and manual groups were below the detection limit, with no detectable residues observed in either group.

#### 3.2.2 Protective effect of robotic compounding on the entire workflow

Surface samples were obtained from finished infusion bags immediately after removal from the compounding chamber to evaluate occupational exposure risks during PIVAS packaging, as well as from the workbench surfaces where these bags were placed 60 min post-unpacking to assess exposure risks during ward administration. Detailed results are presented in Tables 7, 8.

**Table 7:** Comparison of finished product and workbench contamination (cyclophosphamide).

Sampling site	Group	Day 1 (ng/cm^2^)	Day 2 (ng/cm^2^)	Day 3 (ng/cm^2^)	Median (ng/cm^2^)	Range (ng/cm^2^)	Maximum (ng/cm^2^)
Infusion bag surface (immediately post-compounding)	Robotic	0.38907	0.00166	0.00202	0.00202	0.00166 ~ 0.38907	0.38907
	Manual	0.00742	0.02940	0.02062	0.02062	0.00742 ~ 0.02940	0.02940
Workbench (after 60 min)	Robotic	0.0005	0.0005	0.02100	0.0005	0.0005 ~ 0.02100	0.02100
	Manual	0.19913	0.0005	0.00260	0.00260	0.0005 ~ 0.19913	0.19913

LOD = 0.001 ng/cm^2^. Values below the detection limit are all assigned as LOD/2 = 0.0005 ng/cm^2^.

**Table 8:** Comparison of finished product and workbench contamination (fluorouracil).

Sampling site	Group	Day 1 (ng/cm^2^)	Day 2 (ng/cm^2^)	Day 3 (ng/cm^2^)	Median (ng/cm^2^)	Range (ng/cm^2^)	Maximum (ng/cm^2^)
Infusion bag surface (immediately post-compounding)	Robotic	0.003	1.01700	7.17618	1.01700	0.003 ~ 7.17618	7.17618
	Manual	0.07832	1.79476	734.70400	1.79476	0.07832 ~ 734.70400	734.70400
Workbench (after 60 min)	Robotic	0.003	0.003	0.003	0.003	0.003 ~ 0.003	0.003
	Manual	0.003	0.01878	39.62760	0.01878	0.003 ~ 39.62760	39.62760

LOD = 0.006 ng/cm^2^. Values below the detection limit are all assigned as LOD/2 = 0.003 ng/cm^2^.

For cyclophosphamide, at the infusion bag surface (immediately post-compounding) site, the robotic group showed residual values of 0.38907, 0.00166, and 0.00202 ng/cm^2^ over the 3 days, with a median of 0.00202 ng/cm^2^ and a range of 0.00166–0.38907 ng/cm^2^; an high value (0.38907 ng/cm^2^) was observed on Day 1. The manual group showed values of 0.00742, 0.02940, and 0.02062 ng/cm^2^, with a median of 0.02062 ng/cm^2^ and a range of 0.00742–0.02940 ng/cm^2^. Except for a relatively high value observed in the robotic group on Day 1, the residual levels in the manual group were higher than those in the robotic group on the remaining 2 days. At the workbench (after 60 min) site, the robotic group showed values below the detection limit (0.0005 ng/cm^2^) on Days 1 and 2, with a value of 0.02100 ng/cm^2^ on Day 3, yielding a median of 0.0005 ng/cm^2^ and a range of 0.0005–0.02100 ng/cm^2^. The manual group showed a value of 0.19913 ng/cm^2^ on Day 1, a value below the detection limit on Day 2, and 0.00260 ng/cm^2^ on Day 3, with a median of 0.00260 ng/cm^2^ and a range of 0.0005–0.19913 ng/cm^2^. Both groups exhibited considerable variability, with the manual group showing an extreme value (0.19913 ng/cm^2^) on Day 1.

For fluorouracil, at the infusion bag surface (immediately post-compounding) site, the robotic group showed residual values of below the detection limit (0.003), 1.01700, and 7.17618 ng/cm^2^ over the 3 days, with a median of 1.01700 ng/cm^2^ and a range of 0.003–7.17618 ng/cm^2^. The manual group showed values of 0.07832, 1.79476, and 734.70400 ng/cm^2^, with a median of 1.79476 ng/cm^2^ and a range of 0.07832–734.70400 ng/cm^2^. Both groups exhibited the highest values on Day 3, yet the manual group's peak (734.704 ng/cm^2^) markedly exceeded that of the robotic group (7.176 ng/cm^2^), by a factor of approximately 100. At the workbench (after 60 min) site, the robotic group showed values below the detection limit on all 3 days (all 0.003 ng/cm^2^), with a median and range of 0.003–0.003 ng/cm^2^. The manual group showed a value below the detection limit on Day 1 (0.003), followed by 0.01878 and 39.62760 ng/cm^2^ on Days 2 and 3, respectively, with a median of 0.01878 ng/cm^2^ and a range of 0.003–39.62760 ng/cm^2^. The manual group exhibited an extreme value on Day 3, whereas the robotic group consistently showed no detectable residues.

Across both groups, residues detected on infusion bag surfaces and workbenches during packaging and administration stages exceeded those found on operators' gloves during compounding, indicating that occupational exposure risks persist beyond the compounding process. These findings, in conjunction with questionnaire data regarding the protective measures employed by packaging personnel, highlight that PIVAS packaging staff and ward administration nurses constitute an under-recognized population at risk of occupational exposure. Consequently, there is a critical need for enhanced education, training, and supervisory interventions targeting these groups.

#### 3.2.3 Validation of the robot cleaning protocol effectiveness

Samples were collected from key internal functional sites of the device in both the uncleaned state and after cleaning plus 30 min of air circulation to evaluate the effectiveness of the robot cleaning protocol. The detailed results are shown in Tables 9, 10.

**Table 9:** Internal residues of the robotic device (cyclophosphamide) and comparison of cleaning effectiveness.

Sampling site	Group	Day 1 (ng/cm^2^)	Day 2 (ng/cm^2^)	Day 3 (ng/cm^2^)	Median (ng/cm^2^)	Range (ng/cm^2^)	Maximum (ng/cm^2^)
Drug gripper finger (A)	Uncleaned	0.0005	0.0005	0.0005	0.0005	0.0005 ~ 0.0005	0.0005
	Cleaned	0.0005	0.0005	0.0005	0.0005	0.0005 ~ 0.0005	0.0005
Drug loading station (B)	Uncleaned	0.00280	0.00105	0.0005	0.00105	0.0005 ~ 0.00280	0.00280
	Cleaned	0.0005	0.00102	0.0005	0.0005	0.0005 ~ 0.00102	0.00102
Consumable loading gripper (C)—drug side	Uncleaned	0.0005	0.0005	0.0005	0.0005	0.0005 ~ 0.0005	0.0005
	Cleaned	0.0005	0.0005	0.0005	0.0005	0.0005 ~ 0.0005	0.0005
Consumable loading gripper (C)—diluent side	Uncleaned	0.0005	0.0005	0.0005	0.0005	0.0005 ~ 0.0005	0.0005
	Cleaned	0.0005	0.0005	0.0005	0.0005	0.0005 ~ 0.0005	0.0005
Drug preparation table (D)	Uncleaned	0.0005	0.0005	0.0005	0.0005	0.0005 ~ 0.0005	0.0005
	Cleaned	0.0005	0.0005	0.0005	0.0005	0.0005 ~ 0.0005	0.0005
Diluent loading position (E)	Uncleaned	0.0005	0.0005	0.0005	0.0005	0.0005 ~ 0.0005	0.0005
	Cleaned	0.0005	0.0005	0.0005	0.0005	0.0005 ~ 0.0005	0.0005
Waste bin outlet (F)	Uncleaned	0.00122	0.0005	0.00106	0.00106	0.0005 ~ 0.00122	0.00122
	Cleaned	0.00113	0.0005	0.0005	0.0005	0.0005 ~ 0.00113	0.00113

LOD = 0.001 ng/cm^2^. Values below the detection limit are all assigned as LOD/2 = 0.0005 ng/cm^2^.

**Table 10:** Internal residues of the robotic device (fluorouracil) and comparison of cleaning effectiveness.

Sampling site	Group	Day 1 (ng/cm^2^)	Day 2 (ng/cm^2^)	Day 3 (ng/cm^2^)	Median (ng/cm^2^)	Range (ng/cm^2^)	Maximum (ng/cm^2^)
Drug gripper finger (A)	Uncleaned	0.003	0.003	0.003	0.003	0.003 ~ 0.003	0.003
	Cleaned	0.003	0.003	0.003	0.003	0.003 ~ 0.003	0.003
Drug loading station (B)	Uncleaned	0.003	0.003	0.003	0.003	0.003 ~ 0.003	0.003
	Cleaned	0.003	0.003	0.003	0.003	0.003 ~ 0.003	0.003
Consumable loading gripper (C)—drug side	Uncleaned	0.003	0.003	0.003	0.003	0.003 ~ 0.003	0.003
	Cleaned	0.003	0.018	0.003	0.003	0.003 ~ 0.018	0.018
Consumable loading gripper (C)—diluent side	Uncleaned	0.003	0.003	0.003	0.003	0.003 ~ 0.003	0.003
	Cleaned	0.003	0.003	0.003	0.003	0.003 ~ 0.003	0.003
Drug preparation table (D)	Uncleaned	0.003	0.003	0.003	0.003	0.003 ~ 0.003	0.003
	Cleaned	0.003	0.003	0.003	0.003	0.003 ~ 0.003	0.003
Diluent loading position (E)	Uncleaned	0.003	0.003	0.003	0.003	0.003 ~ 0.003	0.003
	Cleaned	0.003	0.003	0.003	0.003	0.003 ~ 0.003	0.003
Waste bin outlet (F)	Uncleaned	0.003	0.003	0.003	0.003	0.003 ~ 0.003	0.003
	Cleaned	0.003	0.003	0.003	0.003	0.003 ~ 0.003	0.003

LOD = 0.006 ng/cm^2^. Values below the detection limit are all assigned as LOD/2 = 0.003 ng/cm^2^.

For cyclophosphamide, at the drug gripper finger (A), consumable loading gripper (C)-drug side, consumable loading gripper (C)-diluent side, drug preparation table (D), and diluent loading position (E) sites, all measurements in both the uncleaned and cleaned groups were below the detection limit (all 0.0005 ng/cm^2^), with no detectable residues observed in either group. At the drug loading station (B), the uncleaned group showed a median of 0.00105 ng/cm^2^ (range: < 0.0005–0.00280), while the cleaned group showed a median of < 0.0005 ng/cm^2^ (range: < 0.0005–0.00102). At the waste bin outlet (F), detectable residues were found on 2 days in the uncleaned group (median: 0.00106 ng/cm^2^; range: < 0.0005–0.00122), compared with only 1 day in the cleaned group (median: < 0.0005 ng/cm^2^; range: < 0.0005–0.00113).

For fluorouracil, at the drug gripper finger (A), drug loading station (B), consumable loading gripper (C)-diluent side, drug preparation table (D), diluent loading position (E), and waste bin outlet (F) sites, all measurements in both the uncleaned and cleaned groups were below the detection limit (all 0.003 ng/cm^2^), with no detectable residues observed in either group. At the consumable loading gripper (C)-drug side site, all three measurements in the uncleaned group were below the detection limit (< 0.003 ng/cm^2^). In the cleaned group, values were also below the detection limit on Days 1 and 3, but a detectable residue (0.018 ng/cm^2^) was observed on Day 2. Apart from this single trace detection, no other residues were found at any site in either group.

In summary, no significant residues were detected at any sampling site before or after cleaning, with the exception of a trace residue (0.018 ng/cm^2^) at the consumable loading gripper (C)—drug side in the cleaned group on Day 2. Overall, the residual drug levels on internal components of the robotic system were extremely low, with no appreciable differences observed between the uncleaned and cleaned groups. Given that the vast majority of samples fell below the detection limit and the sample size was limited (*n* = 3), these descriptive trends should be further validated in future studies.

#### 3.2.4 Cross-contamination risk verification for different drug types with robotic compounding

The potential for cross-contamination arising from robotic compounding processes was systematically investigated. Following the preparation of cytotoxic drugs, carry out standardized cleaning plus 30 min of air circulation, subsequent compounding of non-cytotoxic drugs was performed. Surface samples were obtained from the initial and final infusion bags of the non-cytotoxic drug batches to evaluate the risk of cross-contamination. The comprehensive findings are detailed in Table 11.

**Table 11:** Cross-contamination verification (surface of non-cytotoxic finished infusion bags).

Drug	Group	Day 1 (ng/cm^2^)	Day 2 (ng/cm^2^)	Day 3 (ng/cm^2^)	Median (ng/cm^2^)	Range (ng/cm^2^)	Maximum (ng/cm^2^)
Cyclophosphamide	First bag surface	0.0005	0.0005	0.0005	0.0005	0.0005 ~ 0.0005	0.0005
	Last bag surface	0.0005	0.0005	0.0005	0.0005	0.0005 ~ 0.0005	0.0005
Fluorouracil	First bag surface	0.003	0.003	0.003	0.003	0.003 ~ 0.003	0.003
	Last bag surface	0.003	0.003	0.003	0.003	0.003 ~ 0.003	0.003

Cyclophosphamide LOD = 0.001 ng/cm^2^. Values below the detection limit are all assigned as LOD/2 = 0.0005 ng/cm^2^; Fluorouracil LOD = 0.006 ng/cm^2^. Values below the detection limit are all assigned as LOD/2 = 0.003 ng/cm^2^.

For cyclophosphamide, all measurements on both the first and last bag surfaces were below the detection limit (all 0.0005 ng/cm^2^) over the 3 days, with no detectable residues observed in either group. The median and range were both 0.0005 ~ 0.0005 ng/cm^2^.

For fluorouracil, all measurements on both the first and last bag surfaces were below the detection limit (all 0.003 ng/cm^2^) over the 3 days, with no detectable residues observed in either group. The median and range were both 0.003~0.003 ng/cm^2^.

In summary, no detectable drug residues above the detection limit were observed on either the first or last bag surfaces for both cyclophosphamide and fluorouracil. This finding suggests that, under the present experimental conditions, no detectable cross-contamination was aboserved on the infusion bag surfaces, and no differences were observed between batches (first vs. last). Given that all samples fell below the detection limits and the sample size was limited (*n* = 3), these descriptive findings should be further confirmed in future studies with larger sample sizes.

## 4 Discussion

This three-day comparative study evaluated the occupational protection provided by an intravenous compounding robot across the entire cytotoxic drug workflow, including the compounding, packaging, and administration stages. In parallel, we assessed internal device contamination, cross-contamination risks when the same robotic system was used for subsequent compounding of non-cytotxic drugs, and staff safety awareness. The principal findings are discussed below in relation to the existing evidence base.

### 4.1 Evaluation of occupational protection efficacy of the intravenous compounding robot

#### 4.1.1 Compounding stage

The results of this study demonstrated that during the compounding stage, glove-surface residues of cyclophosphamide and fluorouracil in the robotic group were substantially reduced compared to those in the manual group. This finding is generally consistent with the trend reported by Sessink et al. (9), who also confirmed that robotic systems could reduce operator hand contamination by more than 90%. Such a reduction may be attributed to the fact that the robotic system automated all high-risk manipulations—including ampoule cutting and breaking, vial piercing, and drug solution transfer—within a sealed, negative-pressure environment, thereby minimizing direct contact between personnel and the drugs. Notably, the magnitude of reduction observed in the present study appeared to be even greater, which may be explained by the robotic system's use of integrated pipetting technology that prevented the emission of drug-containing aerosols. In contrast, the systems used in previous studies employed standard syringes for drug aspiration and expelled aerosolized drug residues directly into the environment after each vial transfer, which may partially account for the differences in residual levels observed between the present findings and those reported in the literature. In addition to these technological differences, the lower residual levels observed in our study may also be influenced by differences in sampling conditions and daily compounding workload. In the present study, all sampling was conducted prior to the start of daily operations, after the robotic system and biological safety cabinet had been thoroughly cleaned and disinfected, resulting in a low baseline surface residue. Furthermore, the average daily total compounding volumes at our institution—averaging 1 g for cyclophosphamide and 4 g for fluorouracil—were relatively modest, which likely limited the overall residue burden. These contextual factors should be considered when interpreting the quantitative differences between our findings and those reported in previous studies.

Furthermore, the sporadic but markedly elevated contamination events observed in the manual group in this study—such as the fluorouracil residue on glove surfaces reaching as high as 834.340 ng/cm^2^–suggest that manual operations entail not only routine low-level exposure but also unpredictable high-risk contamination incidents. Owing to its standardized operations and closed-system design, the robotic system may offer a distinct advantage in mitigating such occasional events.

#### 4.1.2 ***Downstream stages of the compounding process — packaging, transport, and*** administration stages

An important finding of this study is that the protective advantage of the robotic system appears not to be confined to the compounding source, but may also extend to downstream stages of the compounding process. Experimental data revealed that during the packaging and administration stages, cyclophosphamide residues in the manual group were approximately 9–10-fold higher than those in the robotic group, while fluorouracil residues reached up to 90 times those detected in the robotic group. Notably, on the surface of finished infusion bags, the manual group exhibited an extremely high residue of 734.704 ng/cm^2^ on Day 3, and this contamination occurred under routine compounding conditions rather than as a result of overt drug spillage. This phenomenon may be associated with the following mechanisms: during manual compounding, repeated piercing of rubber stoppers by syringe needles, as well as ampoule cutting and breaking procedures, may cause microscopic, invisible leakage of drug solution. These trace droplets may adhere to the surfaces of infusion bags or workbenches, and subsequently persist through the packaging, transport, and administration stages. In contrast, the robotic system performs standardized piercing operations within a negative-pressure clean environment, with piercing angle, depth, and frequency precisely controlled by programmed protocols, which may effectively reduce the occurrence of such “invisible leakage”. This finding has important clinical implications, as downstream stages—particularly packaging and drug administration—are often overlooked weak links in occupational exposure protection, a notion reinforced by a paradox observed in our questionnaire survey: while 86.1% of respondents believed that the robotic system reduced occupational hazards at their own work stage, the actual protective behaviors among downstream personnel remained suboptimal−18.2% of packaging staff did not wear gloves, 36.4% did not wear masks, and 45.5% of ward nurses did not consistently wear gloves during drug administration. This discrepancy suggests that the introduction of robotic compounding, while reducing measurable contamination, may inadvertently foster a false sense of security among downstream staff, potentially diminishing their adherence to personal protective measures. In contrast, compounding personnel in our study demonstrated relatively high awareness of occupational protection and consistently adhered to complete protective measures. Given that downstream staff are exposed to quantifiable residues—in some cases exceeding those found on compounding operators' gloves—there is a clear need to extend safety training and supervisory oversight beyond the compounding stage to ensure comprehensive occupational protection across the entire workflow.

### 4.2 Internal residues of the robotic system and cleaning efficacy

Sampling at multiple internal sites of the robotic system showed that drug residues at the vast majority of sites were below the detection limit both before and after cleaning. Only trace residues were detected at the drug loading station (B) and the waste bin outlet (F), with a maximum value of 0.00280 ng/cm^2^, and these residues were further reduced after cleaning. These findings suggest that the internal residual levels of the robotic system after routine use are extremely low, and that the routine cleaning procedure is effective in removing trace residues that are present. This observation is consistent with the possibility that the same robot may be used for subsequent non-cytotoxic drug compounding, although this still requires further confirmation through more rigorous cross-contamination validation studies with larger sample sizes. It is worth noting that a fluorouracil residue of 0.018 ng/cm^2^ was detected at the consumable loading gripper (C)—drug side on Day 2 after cleaning. Although this value is extremely low, it may still indicate potential blind spots in the cleaning procedure, suggesting that further optimization of the cleaning protocol may be warranted in the future.

### 4.3 Cross-contamination risk assessment

The results of the cross-contamination verification experiment showed that no cyclophosphamide or fluorouracil residues were detected on the surfaces of non-cytotoxic finished infusion bags (both the first and last bags), with all samples falling below the detection limits. This finding indicates that, under the experimental conditions of this study, the robotic system, after being used for cytotoxic drug compounding and subsequently cleaned, did not cause detectable drug contamination on the surfaces of finished infusion bags when used for non-cytotoxic drug preparation. This provides preliminary safety data supporting the feasibility of using a shared robotic platform for multiple drug types.

It should be noted, however, that the cross-contamination verification in this study was limited to surface residue detection on infusion bags and did not assess the potential risk of drug contamination of the internal contents of the infusion bags. Future studies should further refine the experimental design to include direct testing of the infusion bag contents and employ more sensitive trace analysis methods.

### 4.4 Limitations and future prospects

Several limitations of this study should be considered when interpreting the findings.

First, the sample size was limited. Only 3 days of sampling data were included per group (*n* = 3). Although the statistical analyses showed that most comparisons did not reach statistical significance, this more likely reflects insufficient statistical power rather than the absence of true differences. Future studies should extend the observation period and increase the sample size to enhance statistical power. Only two cytotoxic drugs—cyclophosphamide and fluorouracil—were evaluated. Future research should broaden the spectrum of investigated agents to include additional cytotoxic drugs such as paclitaxel, cisplatin, and methotrexate, as well as emerging antineoplastic therapies like monoclonal antibodies.

Second, a substantial proportion of measurements fell below the detection limit. Owing to the extremely low residual levels in the robotic group, the majority of sample values were below the method detection limit. In this study, the LOD/2 substitution method was employed for descriptive statistics. Although this approach is a standard practice in environmental monitoring, it inevitably introduces certain assumptions. The use of more sensitive analytical methods in future studies may yield more precise quantitative data.

Third, this was a single-center study. The robotic system, operational procedures, and prescription protocols used in this study were all derived from a single medical institution. The generalizability of the findings therefore needs to be validated through multicenter studies.

Fourth, the Hawthorne effect may have influenced the results. Given that the operators were aware of being under experimental observation, their manual compounding practices may have been more rigorous and cautious than during routine daily operations, thereby potentially reducing the incidence of contamination in the manual group to some extent. Consequently, the experimental data may not fully reflect real-world routine practice.

Fifth, to better sample residues potentially trapped within surface irregularities, cotton swabs were selected as the wipe material. However, due to the porous nature of cotton fibers, the relatively small adsorption area of the swab tip, and the limited solvent volume retained by the swab, this choice may result in a lower recovery rate compared with materials such as filter paper or gauze (19). It should therefore be emphasized that the absolute residue values measured in this study may underestimate true surface contamination, although the positive detections still carry clear occupational exposure warning significance. Importantly, since the same sampling method was applied consistently to both the robotic and manual groups, the relative comparisons between groups remain valid and are unlikely to be affected by these methodological limitations.

Finally, long-term occupational health risks were not assessed. It should be particularly noted that no relevant standards for cross-contamination residues have yet been established in China, nor have supportive reference materials been identified in other countries to date. Therefore, the residue values detected in this study cannot be directly translated into the actual cancer risk for operators. Future research should incorporate epidemiological investigations and exposure assessment modeling to further quantify the health risks associated with occupational exposure.

## 5 Conclusions

In summary, this preliminary study suggests that the intravenous compounding robot demonstrates favorable occupational protective efficacy throughout the entire workflow of cytotoxic drug preparation. Its advantages appear not only to be reflected in the reduction of operator exposure during the compounding stage, but may also extend to downstream stages including packaging, transport, and administration. The internal residual levels of the robot were low and manageable, and the cross-contamination risk appeared low under the present experimental conditions, providing initial feasibility support for its application in multi-drug shared-use scenarios.

However, detectable drug residues on finished infusion bag surfaces and workbenches indicate that packaging and administration staff are also exposed to measurable contamination, especially for the manual group, this risk has received limited attention in previous research. The questionnaire findings further revealed that this downstream risk is compounded by suboptimal adherence to protective measures among non-compounding personnel, highlighting the need for expanded training and supervision across the entire workflow.

The current evidence remains preliminary in nature, and the residue values cannot yet be directly translated into cancer risk for operators. Future studies should expand the sample size, extend the observation period, adopt multicenter designs, and establish exposure–response models linking residual levels to health outcomes, so as to provide higher-level evidence for the development of more evidence-based occupational exposure protection strategies.

## Data Availability

The raw data supporting the conclusions of this article will be made available by the authors, without undue reservation.
